# Noninvasive exploration of Cardiac Autonomic Neuropathy by heart rate and blood pressure variability analysis in Type 2 Diabetic patients

**DOI:** 10.12669/pjms.37.4.3675

**Published:** 2021

**Authors:** Tallat Naz, Noor un Nisa Memon, Kamran Afzal, Ambreen Shakir

**Affiliations:** 1 Tallat Naz, M. Phil Physiology. IBMS, Dow University of Health Sciences, Karachi, Pakistan. SMC, Jinnah Sindh Medical University, Karachi, Pakistan; 2 Noor un Nisa Memon, M. Phil Physiology. SMC, Jinnah Sindh Medical University, Karachi, Pakistan; 3 Kamran Afzal, M. Phil Physiology. College of Medicine, Majmaah University, Al Majma’ah, Saudi Arabia; 4 Ambreen Shakir, MBBS. Ex-lecturer Physiology, Bahria Medical, and Dental College, Bahria University, Karachi, Pakistan

**Keywords:** Cardiac autonomic neuropathy, Diabetic neuropathy, Heart rate variability

## Abstract

**Objectives:**

To determine the prevalence of asymptomatic cardiac autonomic neuropathy and its association with risk factors among patients with Type-2 diabetes mellitus (T2DM)

**Methods:**

The present case-control study was conducted at Department of Medicine, Civil Hospital, Dow University of Health Sciences (DUHS), Karachi, Pakistan during the period September` 2016 to May` 2017. After taking informed consent, subjects from both genders, 72 healthy controls and 72 clinically diagnosed T2DM diabetic patients, age between 30-65 years were selected by non-probability sampling technique. After taking medical history and demographics, Cardiac Autonomic Neuropathy (CAN) was identified by using Ewing`s cardiac autonomic reflex tests (CARTs) and association of risk factors was also investigated.

**Results:**

Severe CAN was identified in 13.9% of T2DM patients while in none of the healthy controls. HR response to deep breathing test was most sensitive (sensitivity= 90%) among all tests. The most common symptoms were Numbness (75.9%) and constipation (69%), resting heart rate and diabetes duration was significantly associated with DCAN.

**Conclusions:**

CAN was highly prevalent in diabetic population that may lead to nephropathy and retinopathy in future. It is highly recommended to use sensitive and simple CARTs in clinics for early detection and early treatment of CAN.

## INTRODUCTION

Diabetes mellitus (DM) is characterized by a high blood sugar level over a prolonged period and its mystifying nature led to pathophysiological alteration in almost all body systems, that causes economic, social and healthcare burden. Diabetes mellitus is a global epidemic that has affected 9.2% of adults globally in 2019.^1^ Studies from Pakistan has depicted that diabetic burden in 2019 was 14.62%^2^ and in 2020 was 13.7%.^3^

Diabetes bring a lot of micro and macrovascular complications after its first onset.^4^ Diabetic cardiac autonomic neuropathy (DCAN) is a microvascular condition where autonomic nerves that control heart rate and blood pressure, become damaged due to hyperglycemia and altered mechanisms such as protein kinase-c, advanced glycation end products, TNF-α signaling and reactive oxygen species.^5^ DCAN remains asymptomatic for several years that ultimately confers morbidity and mortality in these patients.

DCAN is an independent predictor of heart associated mortality and nephropathy, hence it is recommended to perform Cardiac autonomic reflex tests (CARTs) for assessment of subclinical DCAN at first time diagnosis of diabetes and every five years thereafter.^6^,^7^ Subclinical identification aid in better prognosis by timely incorporating treatment to avoid co-morbidities. “Ewing’s CARTs” are Gold standard tests to identify subclinical DCAN that have been used consistently for several decades.^6^,^8^,^9^ It consists of five autonomic reflex tests three of them are based on heart rate changes, and two of them are based on BP changes.^9^ Karachi is among the most populated city in the world, with 9.4% diabetes burden^10^ along with its underlying complications. Literature on this subject is insufficient from Pakistan and this is high time to consider the importance of this devastating complication.

There is no case-control study available from our population, only a few cross-sectional studies are available without utilization of five standard CARTs as recommended by American Diabetic Association. We designed this case-control study to identify and compare the frequency of asymptomatic CAN in Type-II diabetics and healthy controls, by utilizing full set of Ewing`s Gold standard CARTs in Type-II diabetics with no other comorbidities from Karachi, Pakistan.

## METHODS

The present case-control study was carried out at Department of Medicine, Civil Hospital, Dow University of Health Sciences, Karachi, Pakistan from September` 2016 to May 2017. After taking ethical approval from DUHS IRB and Ethics Committee (IRB-508/DUHS/-14 Dated 9-10-2014). Total 144 study participants were included, 72 were T2DM clinically diagnosed cases while 72 were healthy controls, recruited through non-probability purposive sampling technique. Subjects were well informed for study procedure and informed consent was taken from each participant, detailed medical and family history of diabetes and demographics were measured carefully. T2DM patients who were otherwise healthy and with no history of previous or recent heart surgery, cardiac dysfunction, hypertension, apparent neuropathy/ nephropathy/ retinopathy and definite coronary artery dysfunction, were included in the study and vice versa were excluded. Those who were selected for study underwent Cardiovascular Reflex Tests (CARTs) for evaluation of CAN. Tests were performed by same examiner and on same device to avoid biasness in data collection.

### Study protocol:

After initial rest of five minutes, blood pressure was recorded by using Mercury Sphygmomanometer (Yamasu, Japan) and standard 12 lead ECG was recorded by using Power Lab device, Lab Chart Pro-7 software and HRV analysis software module (AD Instruments, Australia), and resting heart rate was automatically computed from lead II ECG recordings. All five Ewing`s cardiac reflex tests designed for the assessment of cardiac autonomic neuropathy were performed according to the procedure previously described in detail (11). Heart rate variability for all three HR tests was measured by HRV analysis module in Lab Chart Pro-7 software.

Ewing`s CARTs consist of (1) *Heart rate response to valsalva maneuver:* HR change was expressed as the Valsalva ratio (mean value taken as normal when ratio was 1.21; borderline if 1.11to 1.20; and abnormal if less than 1.10), (2) *Heart rate response to deep breathing:* The mean value of maximum minus minimum heart rates (beats per minute) for six breathing cycles was considered. (value was taken as normal if it was more than 15 beats per minute, borderline 11-14 beats per minute; abnormal if less than 10 beats per minute), (3) *Heart rate change on immediate standing:* R-R intervals were measured at the 15th and at the 30th beat following beginning to stand. The HR response then expressed as 30:15 ratio (Ratio considered normal if ratio was more than 1.04; borderline if 1.01 to 1.03; and abnormal if ratio is less than 1.00), (4) *Blood pressure response from lying to immediate standing:* The SBP change after two minutes of standing up was measured as the change in response (It was considered normal if less than 10 mmHg; borderline if 11-29 mmHg; abnormal if more than 30 mmHg) and (5) *Blood pressure response to sustained handgrip exercise:* The mean difference highest DBP-value during and before exercise was considered as DPB response to exercise (value is considered normal if it was is more than 6 mmHg; borderline 11-15 mmHg; abnormal if less than 10 mmHg).

Final diagnosis was based on combination of different test results: No CAN; when all five tests were normal or borderline), Early CAN; when only single Heart rate test was abnormal or two border-line, Definite CAN; when any two heart rate tests abnormal, Severe CAN; when any two heart rate tests abnormal plus either of the two Blood pressure tests abnormal.

SPSS-20.0 was used for statistical data analysis. Quantitative data were presented as mean± SD and qualitative data were presented as frequency and percentage. ANOVA and t-tests were performed for quantitative and Chi square tests were performed to compare the categorical variables. Association of CAN with risk factors was identify by Logistic regression analysis. P-value less than 0.05 was considered statistically significant.

## RESULTS

There was a total of 144 subjects (72 cases i.e., T2DM patients and 72 controls), from both genders (103 males and 41 females), respective mean age of controls and cases was 49.69±9.1 and 49.72±8.8 years, and this was not a significant difference (P>0.05). In both groups, mean values of BMI (Kg/m^2^) were normal, yet significantly higher (P<0.05) in cases, with respective values in controls and cases were 22.01±4.3 and 24.78±3.9. Resting HR and resting SBP were significantly while DBP was insignificantly high in cases than controls (Table-I).

**Table-I:** Demographics of study participants.

	CONTROLS	CASES	P value
Subjects	72	72	---
Females	3	38	---
Males	69	34	---
Age (Years)	49.69±9.1	49.72±8.8	>0.05
BMI (Kg/m^2^)	22.01±4.3	24.78±3.9	<0.05
HbA1c	6.50±2.0	9.00±1.53	>0.05
RBS (mmol/l)	7.14±0.38	14.90±4.92	>0.05
FBS (mmol/l)	4.89±0.45	9.024±2.9	>0.05
HR (Beats/min)	73.0±10.4	92.0±11.8	<0.001
SBP (mmHg)	109±13.7	124±11.6	<0.001
DBP (mmHg)	78.0±10.85	81.0±15.6	>0.05

All the values are presented as MEAN ± S.D, P-value showed the level of significance.

All five individual CARTs results showed that frequency of borderline and abnormal results was significantly higher (P<0.05) in cases than controls (Table-II). The overall percentage of CAN after combining all CAN categories in cases, it was found to be 40.3% (early = 13.9, definite =12.5 and severe =13.9), while in control group only early type of CAN was observed in 19.3% (early = 19.3) people. Due to highest sensitivity (90%) of HR response to deep breathing test, it alone identified 36.1% of CAN patients, while Heart rate response from Lying to standing was second most sensitive tests (75% sensitivity) that alone identified 30.5% of CAN patients among all five tests. Glycemic status, mean duration of diabetes, mean age of cases and resting heart rate, all were positively associated with severity of DCAN. Duration of diabetes and resting heart rate were highly significantly correlated (P<0.001) with severe DCAN.

**Table-II:** Comparison of Cardiac Autonomic Functions test results between cases and controls

TESTS	SEVERITY	CONTROLS	CASES	P VALUE
Valsalva Ratio	Normal	68 (94.4%)	57 (79.2%)	<0.01*
	Borderline	4 (5.6%)	11 (15.3%)	
	Abnormal	0 (0%)	4 (5.6%)	
30:15 RR ratio	Normal	70 (97.2%)	50 (69.4%)	<0.001*
	Borderline	0 (0%)	16 (22.2%)	
	Abnormal	2 (2.8%)	6 (8.3%)	
Deep Breathing Test	Normal	68 (94.4%)	46 (63.9%)	<0.01*
	Borderline	4 (5.6%)	12 (16.7%)	
	Abnormal	0 (0%)	14 (19.4%)	
Hand grip Test	Normal	60 (83.3%)	53 (73.6%)	<0.001*
	Borderline	12 (16.7%)	7 (9.7%)	
	Abnormal	0 (0%)	12 (16.7%)	
Postural BP change	Normal	72 (100%)	66 (91.7%)	<0.05*
	Borderline	0 (0%)	4 (5.6%)	
	Abnormal	0 (0%)	2 (2.8%)	

All the values are presented as No. of subjects and percentage, P-value present the level of significance.

## DISCUSSION

Diabetes mellitus and its complications contribute to personal misery as well as constitute an economic burden on the resources of the society. The risk of developing cardiac autonomic neuropathy (CAN) among diabetics is associated with duration and magnitude of hyperglycemia. Usually, CAN showed a diverse spectrum of signs and symptoms, however, in case of diabetes, these manifestations are not apparent because autonomic nerve fibers become damaged and less sensitive to autonomic changes, unfortunately, it keeps silent for several years.^12^ CAN is easily measured by utilizing CARTs at any outpatient facility. Ewing`s non-invasive tests are important because of high sensitivity, specificity, reproducibility, and simplicity for differential diagnosis and to quantify the severity of neuropathy. These Gold standard tests 6,7 are also recommended by American Diabetes Association.^13^ As no routine test is implemented for early detection of CAN in Pakistan, therefore we and few other researchers are working on this co-morbidity of diabetes to highlight its importance.

Although, male participants were more in our study but, frequency of CAN was significantly higher (P<0.05%) in Type-2 diabetic females (44.7%) than males (35.3%). The present study results showed that females are more prone to develop CAN as compared to males. Most recent study from China, also depicted quite high frequency of DCAN, that is 80 out of 386 (20.72%) had DCAN in menopausal T2DM patients.^14^ Age” is one of the confounding factors^15^, but in our study all subjects were age matched so age is not a confounding factor in our study. We found that mean age of Definite CAN cases was 48.67±5.6 and Severe CAN was 50.9±9.39 years, our results are supported by another related study conducted at Faisalabad, Pakistan which showed that mean age of CAN positive diabetics was 52.99±7.00 years.^16^ The average BMI of both groups was normal, however, it was slightly higher in cases than controls (P>0.05), the comparable results were observed in similar studies conducted in India^17^ and in China^18^, when age matched cases and controls were compared, BMI found to be higher in Type-2 diabetics.

Our study is first attempt from Pakistan to perform *case-control* study to identify and compare the frequency of DCAN by utilizing the full set of five CARTs according to the guideline of American Diabetes Association, however, a few cross-sectional studies are available who did not utilize complete set of Ewing’s CARTs. Only one previously published study from Faisalabad, Pakistan, who performed five CARTs in Diabetics to find out the frequency of silent myocardial ischemia in diabetic patients, and it was depicted that even in such a small city of Faisalabad, the prevalence of DCAN was 53.8% with at least two CARTs positive in all patients.^16^

Another study from Hyderabad, Pakistan^19^, utilized three CARTs with some other method, revealed 41.4% prevalence of DCAN. Above mentioned two studies were conducted in a particular region of same ethnic people i.e., Punjab and Sindh, however, our study included almost all ethnics living in Karachi, nevertheless, our results are comparable with these studies and showing significantly higher (P<0.05) prevalence of CAN in cases (40.3%, two or more HR or BP or both tests abnormal) as compared to controls (19.4%, only one HR test abnormal). However, cases had Early, Definite and Severe type of CAN based on combination of several test results, that verify the presence of CAN as per guidelines to professional bodies.^20^ While comparing other countries such as India, CAN is less frequent in Pakistani diabetic population, a recent Indian study depicted 70% of Type-2 diabetics developed CAN.^21^ In the present study, we found that duration of diabetes is a risk factor and showed significant correlation with frequency of CAN, our results are consistent with Yun JS et al.^9^ results. Another risk factor correlated with CAN was poor glycemic status. Although, DCAN show no symptoms but resting tachycardia and constipation found to be shared symptoms.

The present study will contribute considerable insight and data regarding DCAN from population of Karachi, Pakistan. The use of CARTs is not yet implemented in our society, we, appreciate the use of these simple and highly sensitive tests at clinics, at first time diagnosis of Type-2 diabetes and every five years thereafter, as recommended by American and Canadian diabetic associations.

### Study strength and limitations

The strength of our study is that this is a case-control study and results can be compared easily in cases and controls, while in previous cross-sectional studies data from healthy controls was missing. Secondly, we have utilized complete set of Gold standard Ewing`s CARTs recommended by American Diabetes Association, so we are assertive for true results. Though study does not represent whole nation, yet Karachi being a metropolitan city we have data from people of different ethnicities of Pakistan and represented a broader view of this devastating complication of Diabetes.

### Limitation of this study

It is single centered study where most of the patients belong to low- or middle-class socioeconomic status, data from upper class is missing. Multicenter study may represent the actual burden of this disease in Karachi, Pakistan.

## CONCLUSION

Based on the present study findings, it is concluded that to prevent and reverse DCAN, it is important to use CARTs for early and cost-effective diagnosis, to incorporate lifestyle modifications and available therapeutic approaches. We recommend a multi-centered study at provincial level to find actual status of CAN in Pakistan among diabetic population.

### Authors’ Contribution:

**TN:** Conception, research design, data collection, responsible and accountable for the integrity of the work.

**NNM:** Manuscript preparation, Editing and proofreading, Review and final approval.

**KA:** Research protocol, final drafting and review the manuscript.

**AS:** Updated literature search and Statistical analysis, results computations.
